# Supplementary material for: Human plague system associated with rodent diversity and other environmental factors
Source: R Soc Open Sci. 2019 Jun 19;6(6):190216. doi: 10.1098/rsos.190216 (PMC6599787; doi:10.1098/rsos.190216)
Supplement: Supplementary texts, figures and tables [file rsos190216supp1.pdf]

Electronic supplementary material for

Human plague system associated with rodent diversity and other environmental factors

Zhe Sun[a,b,1], Lei Xu[a,b,c,1], Boris V. Schmid[b], Katharine R. Dean[b], Zhibin Zhang[d], Yan Xie[d], Xiye Fang[c], Shuchun Wang[c], Qiyong Liu[c], Baolei Lyu[a], Xinru Wan[d], Jianguo Xu[c,*], Nils Chr. Stenseth[a,b,*], and Bing Xu[a,*]

[a]Ministry of Education Key Laboratory for Earth System Modeling, Department of Earth System Science, Tsinghua University, Beijing 100084, P. R. China;

[b]Centre for Ecological and Evolutionary Synthesis (CEES), Department of Biosciences, University of Oslo, N-0316 Oslo, Norway;

[c]State Key Laboratory of Infectious Disease Prevention and Control, National Institute for Communicable Disease Control and Prevention, Chinese Center for Disease Control and Prevention, Changping, Beijing 102206, P. R. China;

[d]State Key Laboratory of Integrated Management on Pest Insects and Rodents, Institute of Zoology, Chinese Academy of Sciences, Beijing 100101, P. R. China

[1]Z.S. and L.X. contributed equally to this work.

[*]Corresponding authors.

This file includes:

Text
Figures. S1 to S8
Tables S1 to S4
References for electronic supplementary material

Text

1. Average human plague intensity and human population data in China

The first appearance of plague differed among regions [1] and plague outbreaks were often discontinuous, with years of stasis in recorded cases [2]. Thus, we calculated the average plague intensity as the average number of annual human cases when plague occurred (equation 1 in main text).

We used the average human population during the presence years for grids with cases and average human population during 1772 to 1964 for grids without plague cases. This is because the plague appearance were different in time among different grids. We adjusted the human population for grids with plague in case there was a huge change in the grid population from 1772 to 1964. Additionally, we get a better BIC (Bayesian information criterion, accounting by logE in zero-inflated GAM [3]) when we used the different human population for grids with and without cases.

2. Species distribution data in China

The database complied by Xie *et al.* [4] is an aggregation of species distributions from different regions and historical periods (beginning in 1758, see figure S2). We used the whole dataset of the species distribution to calculate species richness, in order to ensure the spatial integrity.

A correlation test was done to examine the data stability in different periods (Table S1) and from different sources. We calculated the correlation coefficients between diversity based on the subsets of the dataset and the whole dataset [4]. We split the dataset by number of records and also truncated a subset by using the peak period of plague (1890 – 1950) in China [5]. We also tested the correlation between the species richness based on an animal distribution survey in 1996 [6] and the aggregated dataset [4]. Table S1 showed that the correlation coefficients were relatively stable, which reflected that the species distribution pattern from different periods and sources was relatively stable.

3. Plague hosts in China

There are 88 species of known plague hosts in China based on field surveys of naturally infected animals and etiological studies [7-9]. The plague hosts are identified based on field surveys of naturally infected animals after 1945, with the criteria of plague infection existing in the host populations [9]. After the field surveys, both animal serological examinations (seroprevalence) and bacteriological examinations (detection of *Yersinia pestis*) were conducted to confirm plague hosts. There are plenty of literatures related to this issue in China, such as Wu *et al.* [10], Li *et al.* [11], Ping [12] and Liu [13]. By combining field surveys and etiological studies, these 88 species are thought to be involved in plague ecosystems and transmission in China, in other words, they have the ability to transmit plague to human [9]. The detailed information of known rodent plague hosts in China was listed in Table S2.

4. Model results

In model with the species richness of all terrestrial vertebrates (model *i*), the annual precipitation was deleted in backward model selection. We found the species richness of terrestrial vertebrates presented a non-significant association with both plague presence and intensity ($p > 0.05$). The human population size in grids exhibited a positive relationship with both plague presence ($\chi^2_{1.94, 5.48} = 32.28$; $p < 0.01$) and intensity ($F_{1.00, 4.00} = 34.37$; $p < 0.01$). There was a positive relationship between plague presence and the local transportation connectivity ($\chi^2_{1.55, 5.48} = 10.53$; $p < 0.05$), but a non-significant result between transportation and plague intensity ($p > 0.05$).

In model with the species richness of all mammals (model *ii*), we found the species richness of mammals had a positive association with plague presence ($\chi^2_{1.00, 7.13} = 7.27$; $p < 0.01$) but a non-significant relationship with intensity ($p > 0.05$). Annual precipitation reflected a positive relationship with plague presence ($\chi^2_{1.89, 7.13} = 8.97$; $p < 0.05$) but a non-significant relationship with plague intensity ($p > 0.05$). The human population size in grids exhibited a positive relationship with both plague presence ($\chi^2_{1.92, 7.13} = 33.55$; $p < 0.01$) and plague intensity ($F_{1.00, 5.00} = 21.39$; $p < 0.01$). There was a positive relationship between

plague presence and the local transportation connectivity ($\chi^2_{1.32, 7.13}$ = 9.35; $p < 0.05$), but a non-significant result between transportation and plague intensity ($p > 0.05$).

In the model with the species richness of rodents (model *iii*), the species richness of rodents had a positive association with plague presence ($\chi^2_{1.81, 7.56}$ = 21.84; $p < 0.01$) and a non-significant association with plague intensity ($p > 0.05$). Annual precipitation reflected a non-significant relationship with plague presence and plague intensity ($p > 0.05$). The human population size in grids exhibited a positive relationship with plague presence ($\chi^2_{1.91, 7.56}$ = 32.16; $p < 0.01$) and plague intensity ($F_{1.21, 5.22}$ = 16.38; $p < 0.01$). There was a positive relationship between plague presence and the local transportation connectivity ($\chi^2_{1.00, 7.56}$ = 5.70; $p < 0.05$), but a non-significant result between transportation and plague intensity ($p >$ 0.05).

In the model with the species richness of known plague hosts (model *iv*), the species richness of known plague hosts had a positive association with plague presence ($\chi^2_{1.49, 7.27}$ = 27.55; $p < 0.01$) but a non-significant association with plague intensity ($p > 0.05$). Annual precipitation reflected a positive relationship with plague presence ($\chi^2_{1.86, 7.27}$ = 7.37; $p <$ 0.05) but a non-significant relationship with plague intensity ($p > 0.05$). The human population size in grids exhibited a positive relationship with plague presence ($\chi^2_{1.93, 7.27}$ = 30.98; $p < 0.01$) and plague intensity ($F_{1.00, 6.14}$ = 18.50; $p < 0.01$). There was a positive relationship between plague presence and the local transportation connectivity ($\chi^2_{1.00, 7.27}$ = 7.70; $p < 0.01$), but a non-significant result between transportation and plague intensity ($p >$ 0.05).

In the model with the species richness of wild rodent plague hosts (model *vi*), the species richness of wild rodents was positively associated with plague presence ($\chi^2_{1.87, 7.60}$ = 43.07; $p < 0.01$; figure S6A), but not with plague intensity in humans ($p > 0.05$; figure S6E). In this model, the precipitation levels had a positive association with both plague presence ($\chi^2_{1.81, 7.60}$ = 10.33; $p < 0.01$; figure S6B) and intensity ($F_{1.56, 5.56}$ = 4.45; $p < 0.05$; figure S6F), as did local human population size (for presence: $\chi^2_{1.91, 7.60}$ = 27.18; $p < 0.01$; figure S6C; for intensity: $F_{1.00, 5.56}$ = 15.63; $p < 0.01$; figure S6G). The local transportation connectivity showed a positive relationship for plague presence ($\chi^2_{1.00, 7.60}$ = 5.78; $p < 0.05$;

figure S6D), and a non-significant positive association with plague intensity ($p > 0.05$; figure S6H).

In the model with the species richness of commensal rodent plague hosts (model *vii*), the transportation connectivity was deleted by backward model selection. We found that the species richness of commensal rodent plague hosts was positively associated with both plague presence ($\chi^2_{1.88,\ 6.73} = 20.77$; $p < 0.01$; figure S6I) and plague intensity ($F_{1.00,\ 4.36} = 5.30$; $p < 0.05$; figure S6L). Annual precipitation reflected a positive relationship with plague presence ($\chi^2_{1.92,\ 6.73} = 11.09$; $p < 0.01$; figure S6J) but a non-significant relationship with plague intensity ($p > 0.05$; figure S6M). The human population size exhibited a positive relationship with both plague presence ($\chi^2_{1.93,\ 6.73} = 37.70$; $p < 0.01$; figure S6K) and intensity ($F_{1.36,\ 4.36} = 21.55$; $p < 0.01$; figure S6N).

We also tested model with the interaction of rodent plague host species richness and transportation connectivity. The formula is as follows,

$$P_i = \mathrm{B}[\alpha + f_{15}(Rh_i) \times Trans_i + f_2(Prec_i) + f_3(Popu_i)] \times \exp[\beta + f_{16}(Rh_i) \times Trans_i + f_6(Prec_i) + f_7(Popu_i) + \varepsilon_i] \quad \text{(S1)}$$

The logE of model (S1) is -884.67, suggesting that keeping individual effect of species richness and transportation in model *v* (logE = -869.33) is better than adding the interaction term.

5. Association strength and significance between diversity and human plague

The best fitting model variant for predicting human plague presence and intensity was model (*v*), which considered the local species richness of rodent plague hosts. The mean and median of rodent plague host diversity in whole of China were 9.14 and 9, respectively. Using 9 as a threshold, we classified the grid cells in China into two categories, high-diversity (species richness of rodent plague hosts greater than 9) and low-diversity groups (species richness of rodent plague hosts less than or equal to 9). Then we tested the association strength and significance between species richness and human plague in these two groups using risk ratio (*RR*) [14] and *chi-square* test [15].

RR is defined as the ratio of plague presence in high-diversity group to low-diversity group, which can be regarded as a measure of the association strength. The frequency of plague presence (plague have occurred) and absence (plague never occurred) in high/low diversity grids was shown in Table S3. Based on Table S3, the ratio of plague presence in high-diversity group (R_h) was 32.31%, while the ratio in low-diversity group (R_l) was 12.86%. The overall ratio (R) was 21.36% for all grids. *RR* was calculated using '*fmsb*' package in R. Finally, *RR* was 2.51 (95%CI: 1.98–3.20; $p < 0.01$), which illustrated that grids with high diversity of rodent plague hosts had 2.51 times the risk of plague presence compared to grids with low diversity.

We also tested the significance of the association using *chi-square* test [15]. The null hypothesis (H_0) was that plague presence is not associated with rodent diversity. If the null hypothesis is true, the expected counts in each category (Table S4) calculated by using the overall ratio R would be close to the observed values. Otherwise, the null hypothesis is not valid under large χ^2.

χ^2 and its associated *p*-value are computed using *chisq.test* function in R, based on the expected and the observed values in Table S3 and Table S4. χ^2 was 60.88 (*d.f.* = 1; $p <$ 0.01) in our study. Thus, the null hypothesis is rejected. The result indicated a significant association between high rodent diversity and plague presence.

6. Spatial sensitivity analysis

In a sensitivity analysis where we merged our grid cells to 2×2 degrees, we found the species richness of rodent plague hosts was still the best predictor of human plague risk (in both presence and intensity). In this coarser-scale model, the species richness of rodent plague hosts still had a positive association with both plague presence ($\chi^2_{1.00, 5.17} = 13.88$; $p < 0.01$; figure S5A) and intensity ($F_{1.00, 5.00} = 5.62$; $p < 0.05$; figure S5E). Annual precipitation reflected a non-significant relationship with plague presence ($p > 0.05$; figure S5B) but a positive relationship with plague intensity ($F_{1.00, 5.00} = 10.31$; $p < 0.01$; figure S5F). The human population size exhibited a positive relationship with plague presence ($\chi^2_{1.00, 5.17} = 5.76$; $p < 0.05$; figure S5C) but a non-significant relationship with intensity ($p >$ 0.05; figure S5G). The local transportation connectivity showed a non-significant

relationship for plague presence ($p > 0.05$; figure S5D) but a positive association with plague intensity ($F_{1.00, 5.00} = 5.75$; $p < 0.05$; figure S5H).

7. Validation of species richness association with human plague

To investigate whether the results were sensitive to any changes in species richness during the recent half century of economic growth and change in China, we calculated the species richness of terrestrial vertebrates, mammals, rodents, known plague hosts, rodent plague hosts, commensal rodent plague hosts and wild rodent plague hosts using only the species distribution records from before 1964. We further replaced the diversity in main text by diversity calculated by truncated records before 1964 (examples in figure S3B and figure S3C). We found that species richness of rodent plague hosts before 1964 had the largest logE compared with other diversity. We also found that the species richness of rodent plague hosts before 1964 had a positive association with both the human plague presence ($\chi^2_{1.84, 6.35} = 12.71$; $p < 0.01$) and intensity ($F_{1.00, 5.32} = 8.01$; $p < 0.01$). These results strengthened the confidence of our findings.

8. Transportation validation

Railways in late 19th century were derived from Wang *et al.* [16]. We used roads in the historical map [17] as the representative main roads in 19th century, because the main road structure was relatively developed in Qing Dynasty and may be relatively stable since then [18]. We subsequently calculated the total length of railways and roads in one-degree grids covering China.

In the model with transportation connectivity in 19th century, we found the species richness of rodent plague hosts had a positive association with plague presence ($\chi^2_{1.89, 7.82} = 39.05$; $p < 0.01$) and intensity ($F_{1.00, 5.76} = 6.04$; $p < 0.05$). Annual precipitation reflected a positive relationship with plague presence ($\chi^2_{1.88, 7.82} = 8.41$; $p < 0.05$) and a non-significant association with intensity ($p > 0.05$). The local human population size exhibited a positive relationship with plague presence ($\chi^2_{1.92, 7.82} = 30.02$; $p < 0.01$) and intensity ($F_{1.42, 5.76} = 13.06$; $p < 0.01$). The transportation connectivity showed a non-significant positive association with plague presence and intensity (presence: $p < 0.1$).

Figure S1. Zero-inflation test of log-transformed average plague intensity in China.

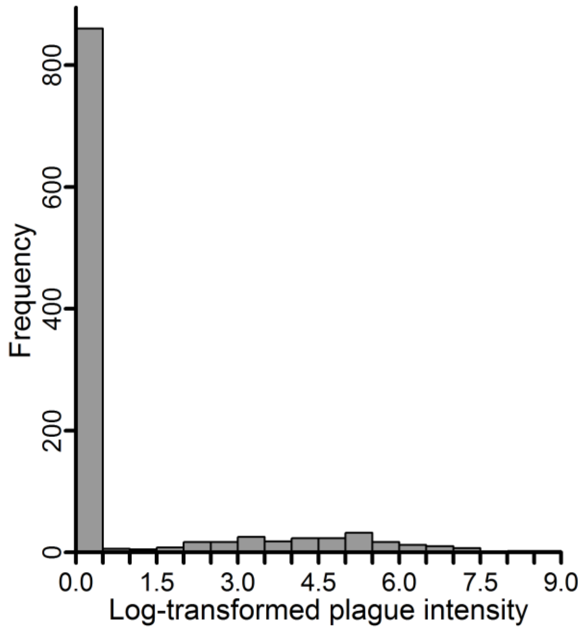

Figure S2. Histogram of the number of records in the species distribution data in China.

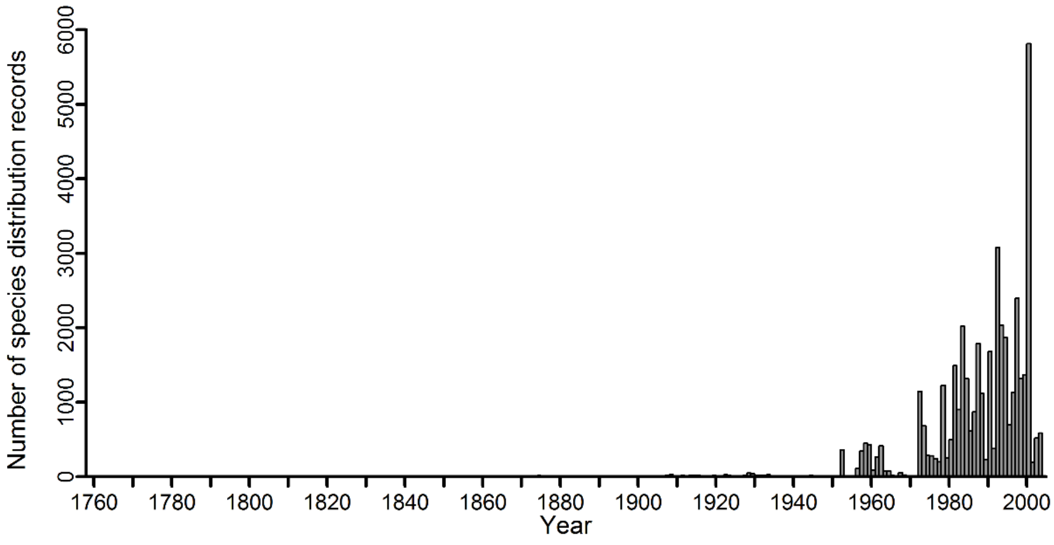

Figure S3. Spatial distribution of species richness in mainland China. (A) Species richness of terrestrial vertebrates. **(B)** Species richness of terrestrial vertebrates, using species distribution records before 1964. **(C)** Species richness of rodents, using species distribution records before 1964.

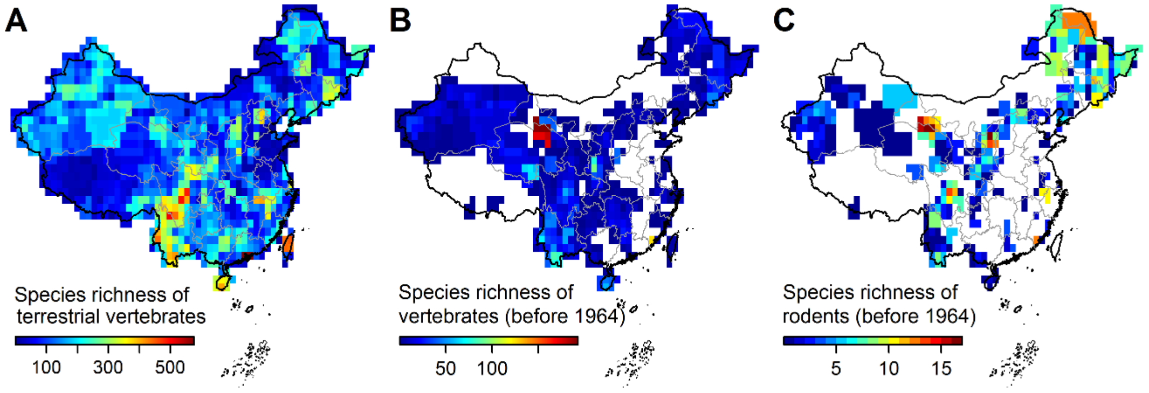

Figure S4. Semi-variogram of model residuals. The x-axis is the grid distance and the y-axis is the semi-variance. These plots indicate that there is some spatial correlation in the residuals between adjacent grid cells, with a semi-variogram score of 0.76-0.79 between plots A-C. **(A)** Model *v*. **(B)** Model *vi*. **(C)** Model *vii*.

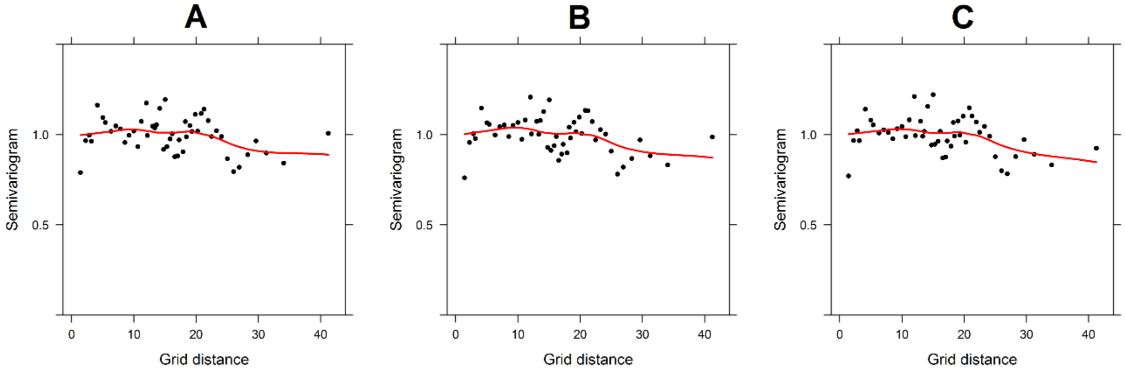

Figure S5. Spatial sensitivity analysis of model (*v*) with species richness of rodent plague hosts by merging grids to 2-degree grids. (A-D) The binomial part quantifies the presence of plague. **(E-H)** The lognormal part evaluates the positive plague intensity.

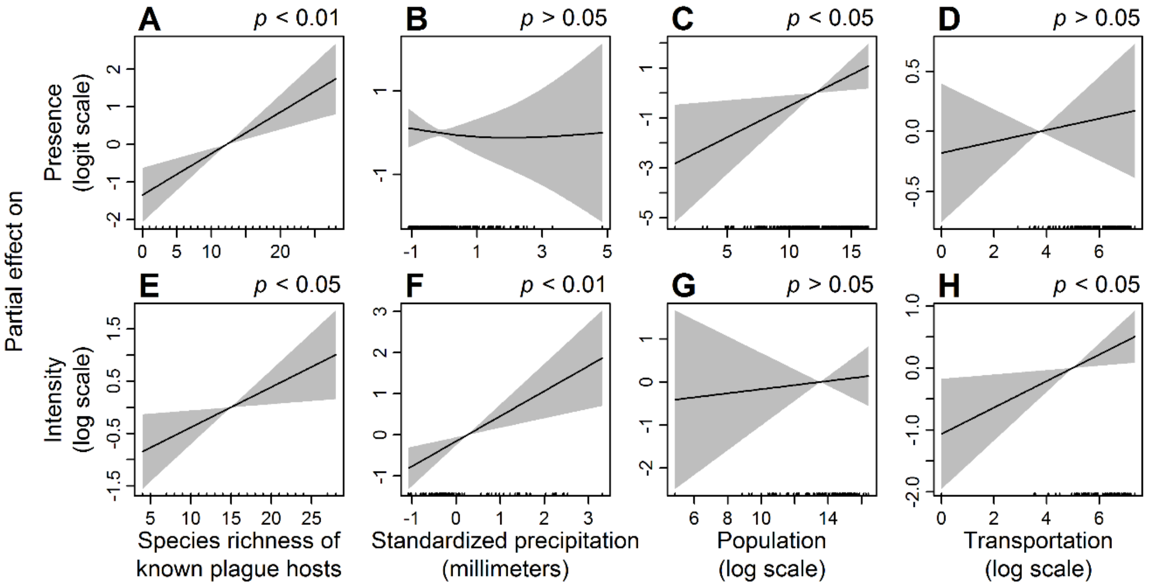

Figure S6. Partial effects of additive parts on human plague presence and intensity of the model (*vi*) with species richness of wild rodent plague hosts, and model (*vii*) with species richness of commensal rodent plague hosts. (A-H) Results of model with species richness of wild rodent plague hosts, where **(A-D)** quantify the presence of plague and **(E-H)** evaluate the positive plague intensity**. (I-N)** Results of model with species richness of commensal rodent plague hosts, where **(I-K)** quantify the presence of plague and **(L-N)** evaluate the positive plague intensity.

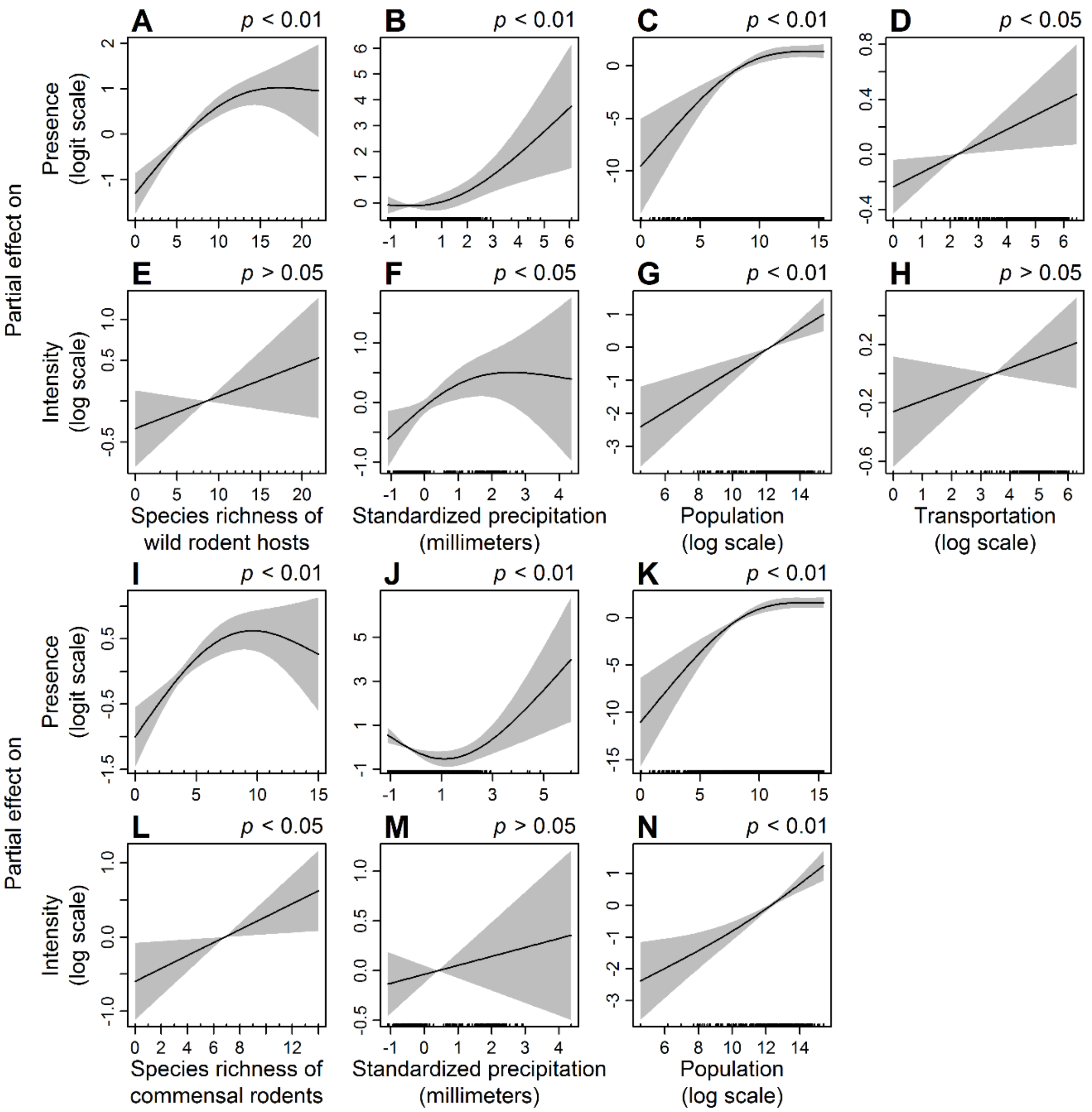

Figure S7. The proportion of species richness of wild/commensal rodent plague hosts to rodent plague hosts in mainland China. (A) Ratio of wild rodent hosts to all rodent plague hosts. **(B)** Ratio of commensal rodent hosts to all rodent plague hosts. The dotted line in figures refers to the Heihe – Tengchong Line [19] which is a demarcation based on the distribution of human population in China. **(C)** Locations of infected villages in China during the Third Pandemic and central part of China.

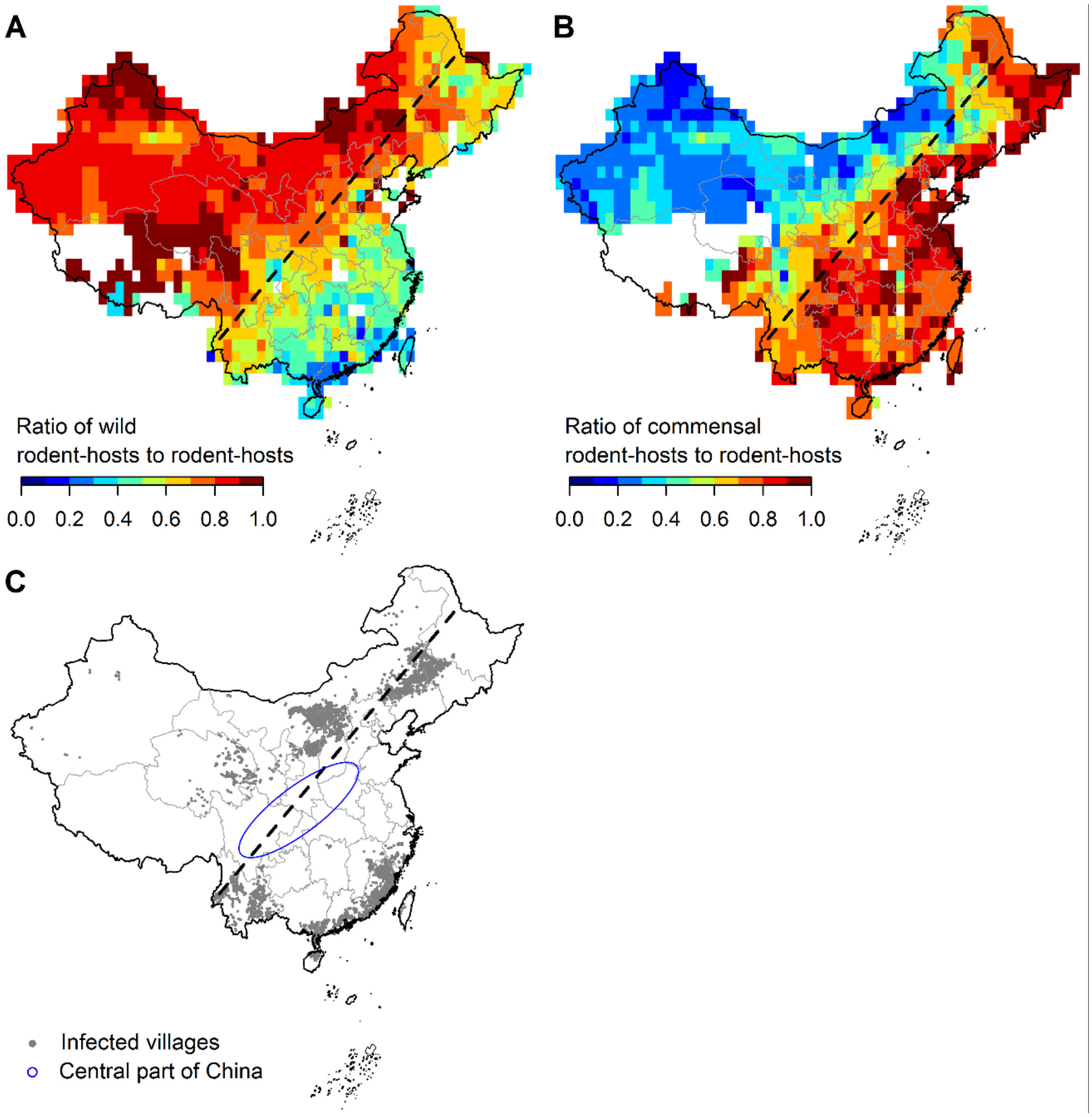

Figure S8. Transportation routes in late 19th century.

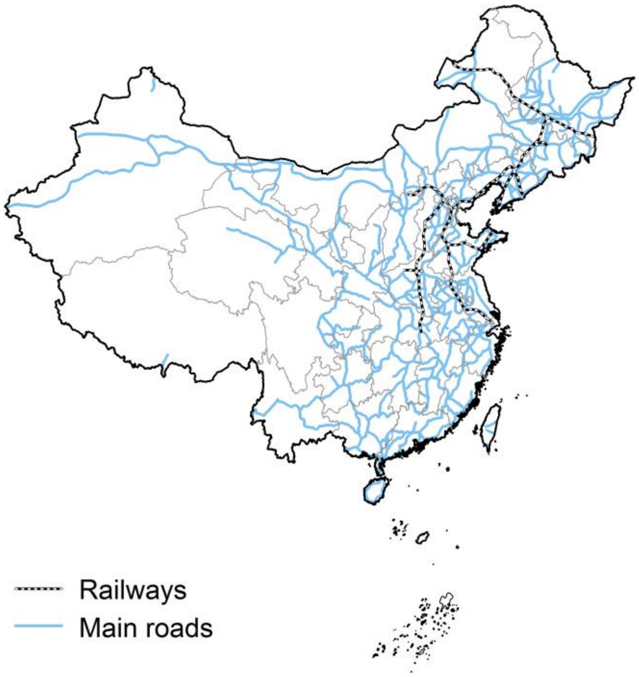

Table S1. Correlation test between terrestrial vertebrate diversity in different periods and the terrestrial vertebrate diversity of the whole dataset. Species diversity in 1996 was calculated based on an animal distribution survey.

ID	Time period	Number of records	Correlation coefficient
1	1890 - 1950	544	0.31*
2	before 1983	10574	0.48*
3	1983 - 1992	10006	0.43*
4	1993 - 1998	11201	0.46*
5	1999 - 2004	9806	0.39*
6	1996	10905	0.50*

* Significant ($p < 0.05$).

Table S2. Lists of rodent plague hosts in China and classification of rodent plague hosts according to their habitat. Information of rodent habitats were derived from Yang and Yan [20] and Pan *et al.* [21].

No.	Name	Habitats	Classification
1	*Marmota baibacina*	Prairie	Wild
2	*Marmota caudata*	Prairie	Wild
3	*Marmota himalayana*	Prairie	Wild
4	*Marmota sibirica*	Prairie	Wild
5	*Sciurotamias forresti*	Forest	Wild
6	*Spermophilus alashanicus*	Prairie, desert	Wild
7	*Spermophilus dauricus*	Prairie, desert	Wild
8	*Spermophilus pallidicauda*	Prairie, desert	Wild
9	*Spermophilus undulatus*	Prairie, forest	Wild
10	*Callosciurus erythraeus*	Forest	Wild
11	*Dremomys pernyi*	Forest	Wild
12	*Tamiops swinhoei*	Forest	Wild
13	*Allactaga sibirica*	Prairie, desert	Wild
14	*Cardiocranius paradoxus*	Prairie, desert	Wild
15	*Dipus sagitta*	Desert, prairie	Wild
16	*Stylodipus andrewsi*	Desert	Wild
17	*Myospalax aspalax*	Prairie	Wild
18	*Alticola argentatus*	Prairie	Wild
19	*Alticola semicanus*	Prairie	Wild
20	*Ellobius tancrei*	Prairie, desert	Wild
21	*Eolagurus przewalskii*	Prairie, desert	Wild
22	*Eothenomys miletus*	Forest, farmland	Wild/Commensal
23	*Lasiopodomys brandtii*	Prairie	Wild
24	*Lasiopodomys fuscus*	Prairie	Wild
25	*Microtus gregalis*	Prairie	Wild
26	*Microtus limnophilus*	Prairie	Wild
27	*Allocricetulus eversmanni*	Prairie, desert	Wild
28	*Cricetulus barabensis*	Prairie, desert, farmland	Wild/ Commensal
29	*Cricetulus migratorius*	Prairie, desert, forest, farmland	Wild/ Commensal
30	*Phodopus campbelli*	Desert, prairie	Wild
31	*Phodopus roborovskii*	Desert, prairie	Wild
32	*Tscherskia triton*	Farmland, prairie	Wild/ Commensal
33	*Meriones meridianus*	Desert	Wild
34	*Meriones unguiculatus*	Prairie, farmland, desert	Wild/ Commensal
35	*Rhombomys opimus*	Desert	Wild

36	*Apodemus agrarius*	Farmland, forest	Wild/ Commensal
37	*Apodemus chevrieri*	Farmland, forest	Wild/ Commensal
38	*Apodemus draco*	Forest, farmland	Wild/ Commensal
39	*Apodemus latronum*	Forest, farmland	Wild/ Commensal
40	*Apodemus peninsulae*	Forest, farmland	Wild/ Commensal
41	*Bandicota indica*	Farmland	Commensal
42	*Micromys minutus*	Farmland	Commensal
43	*Mus caroli*	Farmland	Commensal
44	*Mus musculus*	Residence, farmland	Commensal
45	*Niviventer andersoni*	Forest	Wild
46	*Niviventer confucianus*	Forest, farmland	Wild/ Commensal
47	*Niviventer fulvescens*	Forest, farmland	Wild/ Commensal
48	*Rattus rattus*	Residence, farmland	Commensal
49	*Rattus losea*	Farmland	Commensal
50	*Rattus nitidus*	Residence, farmland	Commensal
51	*Rattus norvegicus*	Residence, farmland	Commensal
52	*Rattus tanezumi*	Residence, farmland	Commensal
53	*Cavia porcellus*	Residence	Commensal
54	*Myodes proditor*	Forest	Wild

Table S3. The observational frequency of plague presence in high-diversity and low-diversity grid cells.

	Presence of plague (number of grids)	Absence of plague (number of grids)	Sum	Ratio of plague presence
High diversity	158	331	489	32.31%
Low diversity	81	549	630	12.86%
Sum	239	880	1119	21.36%

Table S4. The expected frequency of plague presence in high-diversity and low-diversity grid cells.

	Presence of plague (number of grids)	Absence of plague (number of grids)
High diversity	104.45	384.55
Low diversity	134.57	495.43

Reference

1. Xu L., Stige L.C., Kausrud K.L., Ari T.B., Wang S., Fang X., Schmid B.V., Liu Q., Stenseth N.C., Zhang Z. 2014 Wet climate and transportation routes accelerate spread of human plague. *Proc Biol Sci* **281**(1780), 20133159. (doi:10.1098/rspb.2013.3159).
2. Institute of Epidemiology and Microbiology, Chinese Academy of Medical Sciences. 1980 *The History of Spread of Plague in China*. pp. 33-1794. Beijing, People's Medical Publishing House.
3. Liu H., Chan K.-S. 2010 Introducing COZIGAM: an R package for unconstrained and constrained zero-inflated generalized additive model analysis. *J Stat Softw* **35**(11), 1-26. (doi:10.18637/jss.v035.i11).
4. Xie Y., Zhang S., Wang W. 2009 *Geographical Atlas of Biodiversity in China*. pp. 1-246. Changsha, Hunan Education Press.
5. Ben-Ari T., Neerinckx S., Agier L., Cazelles B., Xu L., Zhang Z., Fang X., Wang S., Liu Q., Stenseth N.C. 2012 Identification of Chinese plague foci from long-term epidemiological data. *Proc Natl Acad Sci USA* **109**(21), 8196-8201. (doi:10.1073/pnas.1110585109).
6. China's State Forestry Administration. 2009 *Survey of Key Terrestrial Wildlife Resources in China*. pp. 1-348. Beijing, China Forestry Publishing House.

7. Fang X. 1990 *Natural foci of plague in China*. pp. 1-290. Beijing, People's Medical Publishing House.

8. Wang Y., Liu Q., Cong X., Xu C., Li Y. 2007 Plague reservoirs and their classification in natural foci of China. *Chin J Vector Biol Control* **18**(2), 127-133.

9. Qin C., Xu L., Zhang R., Liu Q., Li G., Fang X. 2012 Ecological-geographic landscapes of natural plague loci in China V. Biological characteristics of major natural reservoirs of Yesinia pestis. *Chin J Epidemiol* **33**(8), 692-697.

10. Wu L., Chen Y.H., Pollitzer R., Wu C.Y. 1936 *Plague: a Manual for Medical and Public Health Workers*. pp. 1-421. Shanghai, Weishengshu National Quarantine Service.

11. Li C., Zheng Y., Wang H., Wang Z., Wang L., Wei S., Cui B., Wang G., Chen H., Qi Z., et al. 2009 Investigation on the space structure of plague natural foci in the Sanjiangyuan area in Qinghai Province. *Chin J Endemiol* **28**(5), 522-526.

12. Ping C. 2013 Analysis of plague monitoring results in Lhasa, Tibet during 1988 and 2011. *Chin J Endemiol* **32**(4), 452-452.

13. Liu Y. 2000 *The atlas of plague and its environment in the People's Republic of China*. pp. 48-79. Beijing, Science press.

14. Porta M. 2014 *Dictionary of epidemiology*. pp. 245-252. Oxford, England, Oxford University Press.

15. Fisher R.A. 1922 On the Interpretation of χ2 from Contingency Tables, and the Calculation of P. *J R Stat Soc Series B Stat Methodol* **85**(1), 87-94. (doi:10.2307/2340521).

16. Wang J., Jin F., Mo H., Wang F. 2009 Spatiotemporal evolution of China's railway network in the 20th century: An accessibility approach. *Transp Res A Policy Pract* **43**(8), 765-778. (doi:10.1016/j.tra.2009.07.003).

17. Yang J., Li Q., Qiu Z. 1946 *Map of China*. p. 5. Shanghai, Huancheng Press.

18. Committee of history of road in ancient China. 1994 *History of Road in Ancient China*. Beijing, China Communications Press.

19. Hu H. 1935 The Distribution of Population in China, with Statistics and Maps. *Acta Geogr Sin* **15**(2), 33-74.

20. Yang Q., Yan K. 2007 *Animal Color Map in China*. pp. 1-331. Beijing, Science Press.

21. Pan Q., Wang Y., Yan K. 2007 *Color Atlas of Chinese Mammals*. pp. 1-420. Beijing, China Forestry Publishing House.
